# Electroconductive Polymer Repellent Composites Based on *N*,*N*-Diethyl-3-methylbenzamide

**DOI:** 10.3390/molecules30051036

**Published:** 2025-02-24

**Authors:** Sergei Zverev, Daria Savraeva, Yulia Ignatova, Victoria Aristova, Leonid Martynov, Konstantin Sakharov, Valeriya Dubinich, Sergei Andreev

**Affiliations:** 1 Disinfectology Institute of F.F. Erisman FSCH of Rospotrebnadzor, 18 Nauchny Drive, Moscow 117246, Russia; 2 MIREA–Russian Technological University (M.V. Lomonosov Institute of Fine Chemical Technologies), 86 Vernadsky Prospekt, Moscow 119571, Russia; 3 School of Materials Science and Engineering, Nanyang Technological University (NTU), 50 Nanyang Avenue, Singapore 639798, Singapore; 4 Facility for Analysis, Characterization, Testing and Simulation (FACTS), Nanyang Technological University (NTU), 50 Nanyang Avenue, Singapore 639798, Singapore; 5 Gubkin University, 65 Leninsky Prospekt, Moscow 119991, Russia

**Keywords:** conductive polymer, repellent, DEET, multi-walled carbon nanotubes, impedance, thermal diffusion, mosquitoes

## Abstract

In this study, electrically conductive polymer composites based on repellent *N*,*N*-diethyl-3-methylbenzamide with concentrations ranging from 6 to 30 wt% were developed. The electrical resistivity of repellent composites, as determined by electrochemical impedance spectra, ranges from 150 to 171 Ohm, which allows such materials to be used when a low voltage is applied. The study of the rheological properties of the obtained repellent composites and the analysis of the TGA curves demonstrated that the dynamic viscosity of the materials has a significant effect on the thermal diffusion of the repellent. The study of the thermal diffusion of *N*,*N*-diethyl-3-methylbenzamide demonstrated that a higher yield of repellent (up to 36.4 × 10^−8^ mol) is achieved when the material is applied in the form with the shortest conductor length of 14 mm. The graphs showing the relationship between the electrical flux and the concentration of *N*,*N*-diethyl-3-methylbenzamide, which was calculated via the Peltier and Thompson equations, show that, according to Onsager’s theory, the total flux of the substance is highest when a voltage is applied to the material with the shortest conductor length. Thus, the developed repellent composite is a promising material for protection against blood-sucking insects.

## 1. Introduction

Conductive polymer composites represent a new class of materials that have been used in various fields, including biomedicine, electronics and agriculture [1,2,3,4,5]. Due to their unique mechanical, physical and biological properties, conductive polymers are highly suitable for use as materials for skin regeneration [3], drug delivery systems and biosensor carriers [4]. Of particular interest are polymer materials with electrical properties that allow electron transfer at low voltages. The components that determine electrical conductivity are polyaniline, polypyrrole, polythiophene [4,5], metal oxides and nanoparticles, and carbon compounds of various modifications, such as graphene, fullerene and carbon nanotubes [6,7,8]. The addition of multi-walled carbon nanotubes results in materials with low resistivity, enabling them to function at lower power. Moreover, the creation of flexible and ultrathin structures allows the production of materials with a wide range of geometric shapes, thereby expanding their potential applications [9].

Polymer functional materials are also widely used in agriculture [10]. In addition to the benefits of using such materials to improve the growth conditions of agricultural products, such as controlled release mechanisms, targeted photosynthesis and delivery systems, an important property of functional polymers is protection against pests [11]. Statistics indicate that the annual economic impact of insect pests in the agricultural sector exceeds 470 billion dollars, resulting in crop damage amounting to 20% [12]. The careful application of insecticides, in doped or conjugated forms, is a proven strategy for mitigating the risk of crop destruction.

Polymer composites have also been used to protect people from attacks by dangerous blood-sucking insects [13]. At present, mosquitoes are a serious threat to public health because they are carriers of yellow fever, West Nile fever, tularemia, malaria, and parasitosis. Given the challenging epidemiological situation in humid and temperate regions and the increasing resistivity of insects to insecticides, there is an increasing focus on the research and development of functional composites [14,15,16]. Such approaches have the potential to allow more effective and targeted use of existing active ingredients, which can help reduce the risk of insect resistivity. At the same time, it could be possible to avoid successive increases in the concentration of the applied substances, which could inevitably lead to environmental pollution.

One approach to creating composites to protect against blood-sucking insects involves the use of polymers, which are electrically conductive materials with repellent properties. Previous studies described approaches for producing polymer composites with pronounced electrically conductive properties, and it has been demonstrated that a conductive material with low resistivity can be obtained by doping it with carbon nanotubes [17]. In addition, polymer materials with added repellents have also been studied previously [18]. However, to achieve the target effect on blood-sucking insects, it is necessary to develop a functional composite where controlled release can be achieved by external influences, e.g., low voltage action.

In this study, we investigated electrically conductive polymer composites based on multi-walled carbon nanotubes, hydroxyethyl cellulose polymer and the repellent *N*,*N*-diethyl-3-methylbenzamide, known as DEET. Recently, there has been great interest in studying the properties of composite materials under the influence of Joule’s heat [19] and the efficacy of DEET against blood-sucking insects has been proven in previous studies [20,21]. The obtained repellent composites were analyzed via electrochemical impedance and thermogravimetric analysis methods. The rheological properties and thermal diffusion of the repellent at different application rates were also studied.

## 2. Results and Discussion

### 2.1. Preparation of a Repellent Composite Material

To create an electrically conductive DEET-based composite material, it was crucial to select components that would allow the controlled release of the repellent under low voltage, i.e., that the composite would have low electrical resistance. The formation of a closed electrical target heats the material via Joule heat, releasing the repellent. To achieve this effect, a polymer carrier must be selected for the repellent to prevent uncontrolled release of the substance from the material. The selection of hydroxyethylcellulose (waterless) as the polymer substance was predicated on its ability to act as a binding agent for the other components of the system, including DEET. This is due to its recognized capacity as a thickening agent, with the additional advantage of its ability to dissolve in polar organic solvents. Dimethyl sulfoxide (DMSO) was chosen as the solvent for both the polymer and the DEET repellent due to of its ability to enhance the conductivity of the electrical current and low solubility in water, which is important for effective dissolution of DEET [22]. The selection of the electroconductive agent for the composite material was carried out by preparing a composition containing 30 wt% DEET, 1 wt% hydroxyethyl cellulose, 58.9 wt% DMSO, and 0.1 wt% ethylene glycol with the addition of electroconductive compounds of different contents. The compounds selected for investigation were activated carbon, copper nanoparticles, polyaniline with added silver particles (PANI/Ag) and multi-walled carbon nanotubes. Following the preparation of the mixtures, the composite was applied to a 50 × 20 × 1 mm substrate. The results of the measurements are summarized in Table 1.

A comparison of the electrical resistivity values of the obtained mixtures revealed that multi-walled carbon nanotubes with a content of 10 wt% presented optimal conductive properties and low resistivity. The electrical resistivity of this composite was measured at 140 ± 28 Ohms, which allows it to conduct a low voltage current. In all other cases, the electrical resistivity was several orders of magnitude greater, which did not allow the use of low voltage when working with composites. To further investigate the properties of the repellent electrically conductive composite, five samples with DEET content of 6–30 wt% (according to Section 3.2.1 in the Section 3) were prepared.

### 2.2. Measurement of the Impedance Spectra of a Repellent Composite Material

**Figure 1:**
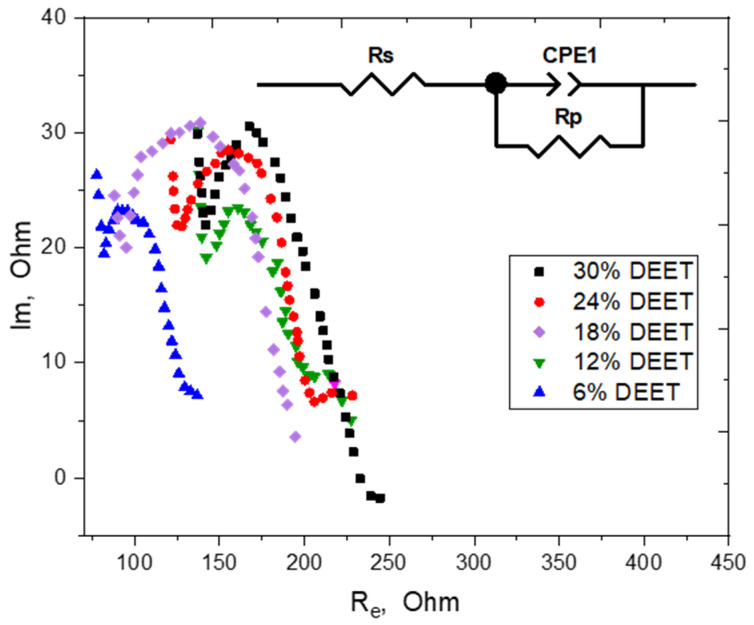
Nyquist diagrams of repellent composite material with different DEET content were recorded in a 2.5 mmol/L K_3_Fe(CN)_6_/K_4_Fe(CN)_6_ solution in 0.1 mol/L KCl. The AC voltage amplitude was set at 10 mV, and the frequency ranged from 1 Hz to 13 kHz. Inset: Randles equivalent electrical circuit (EEC).

To confirm the resistivity values of the repellent composite material based on multi-walled carbon nanotubes, impedance analysis was carried out to determine the resistivity to alternating current resulting from the combined action of resistivity and reactance in a circuit [23]. Electrochemical impedance spectroscopy (EIS) was used to determine the resistivity and capacitance of the repellent composite samples via an electroactive ferricyanide/ferricyanide redox couple as an electrochemical probe. The results of the impedance measurements are presented as Nyquist diagrams in Figure 1.

In the high frequency region, the Nyquist diagrams take the form of a semicircle, the radius of which corresponds to the charge transfer resistivity at the composite/solution interface. According to the data presented thus far, the most suitable model for describing surface phenomena in a multi-walled carbon nanotube-based composite is the generalized equivalent electrical circuit (EEC) of Randles [23]. The EEC consists of an uncompensated resistivity with a parallel combination of the charge transfer resistivity (Rp) and the constant phase element (CPE1). The primary contribution to the uncompensated resistivity is the solution resistivity (Rs). The resistivity Rp reflects the electron transfer complexity of the electrochemical probe ([Fe(CN)_6_]^3−/4−^) between the solution and the electrode. The constant phase element models the inhomogeneity of the surface or bulk characteristics of the material under investigation.

As illustrated in Figure 1, an increase in the DEET content of the composite leads to an expansion of the impedance hodograph semicircle, signifying an elevated resistivity to electron transfer. The diagrams obtained were approximated in accordance with Randels EEC, and the results of this approximation are outlined in Table 2.

The data processing results indicated that the repellent composite material, with varying concentrations of DEET, exhibited a resistivity (Rp) ranging from 150 to 171 Ohm. This finding is generally consistent with the measurements obtained through the utilization of a multimeter. It can be concluded that the presence of the DEET repellent in the aforementioned concentration range does not significantly affect the electrical conductivity of the material in its entirety. Furthermore, the composite itself is characterized by a high degree of homogeneity. Thus, the data obtained from the EIS measurements allow estimation of the electrical conductivity of the carbon composites and are in agreement with previously reported results.

### 2.3. Measurement of the Voltametric Properties of the Repellent Composite Material

**Figure 2:**
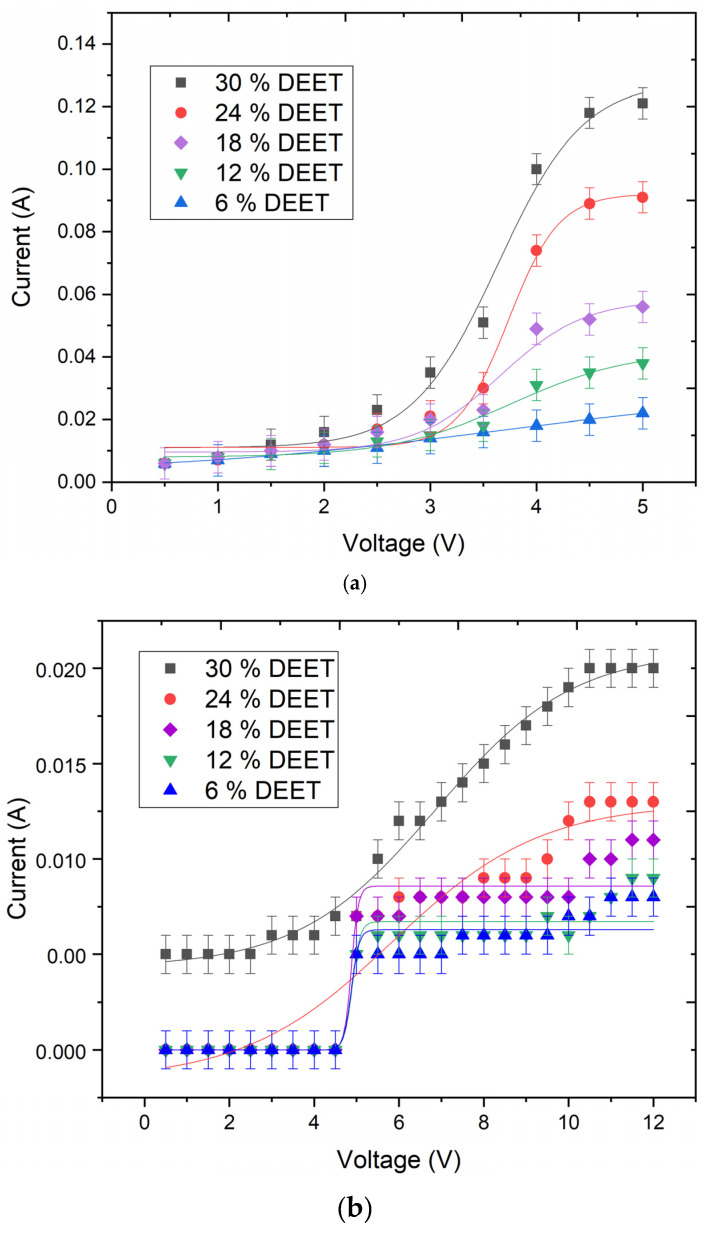

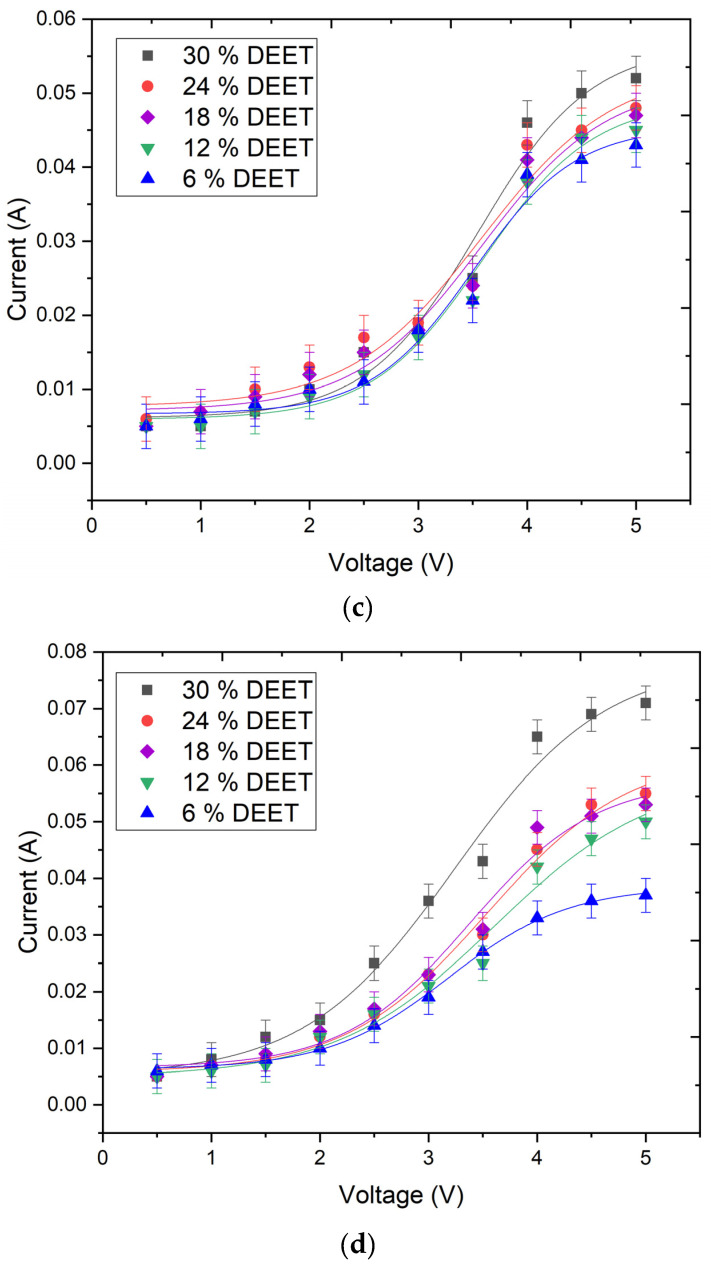

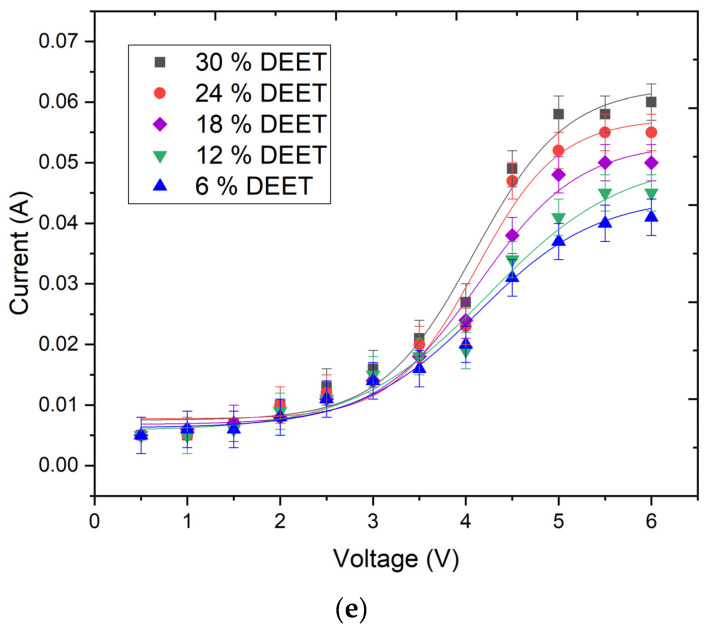
Plots of resistivity measurements of repellent composites with different DEET content in different geometric shapes: straight strip (**a**), meander (**b**), and grids (**c**–**e**) (n = 10).

The determination of the voltametric properties of conductive composites is a key factor in the prediction of the performance of materials with controlled release of active ingredients. The voltametric properties of the repellent composites with different DEET content were determined through experimentation with model substrates (according to Section 3.2.3 in the Section 3). These model substrates were formed from straight strips (a), meandering strips (b), and grids (c–e). An electric current was supplied to these substrates in different ways. The selected models demonstrate the most effective geometrical shapes for the developed composite (Figure 2 and Table 3).

The data obtained indicate that, across all the geometries, the composite containing 30 wt% DEET exhibited superior electrical conductivity compared with the other compositions (Table 3). However, the variation in resistivity values between compositions is not statistically significant. In addition, the geometric shape of the model in which the repellent composite is applied is particularly important in determining the conductivity of the electrical charge. To illustrate this point, consider the material applied in the meander shape. To initiate conduction of electric current, a voltage of more than 5 V is needed. In contrast, a voltage of 2 V is sufficient for other shapes. Conversely, when applied as films with a thickness of 1 mm, the obtained composites are incapable of conducting voltages above 6 V (or 12.5 V for the meander shape), due to significant overheating of the conductor, which subsequently leads to breakdown of the electrical circuit. Consequently, for each geometric shape in which the repellent composites were positioned, a ’voltage range’ was delineated, and further investigations were conducted within this range.

### 2.4. Measurement of Rheological Properties and Thermogravimetric Analysis of the Repellent Composite Material

**Figure 3:**
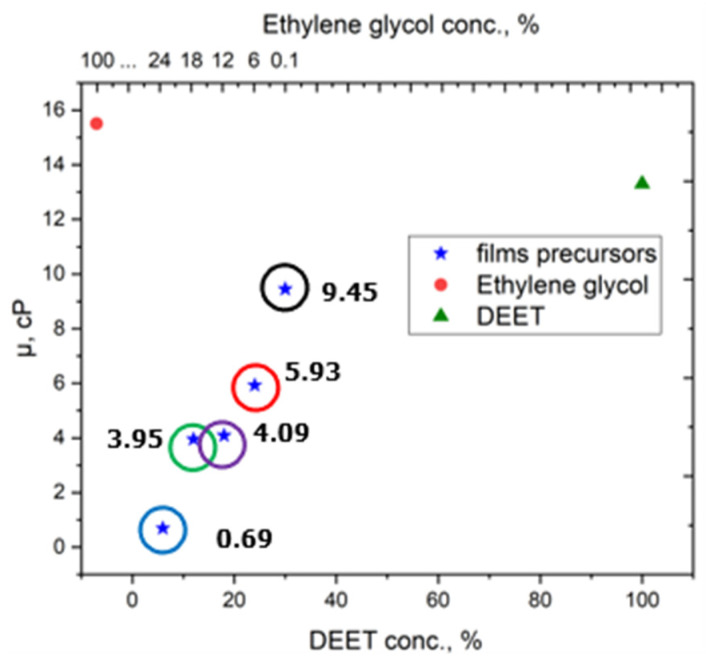
Dynamic viscosity of repellent precursors as a function of DEET concentration.

The rheological properties of electrically conductive composite materials with different DEET repellent content can also have a key influence on the controlled release of the substance from the polymer matrix. As part of our research, we studied how the initial DEET concentration affects the composite’s physicochemical and electrical properties. For this purpose, ethylene glycol was utilized as a substitute for DEET, a compound that has been extensively researched and is well-documented [24]. The dynamic viscosity value of ethylene glycol is the closest to that of the DEET repellent among all known compounds of similar rheology. The dynamic viscosity indices (cP) of the repellent precursors with varying DEET concentrations (6–30 wt%) were determined, and the data obtained were then compared, as illustrated in Figure 3.

Despite the similar values of the dynamic viscosities of DEET and ethylene glycol (13.3 and 15.5 cP, respectively), the viscosities of the obtained compositions differ. This is to be expected and has a significant effect on the electrical conductivity of the repellent composites, which is crucial for the thermal diffusion of DEET.

**Figure 4:**
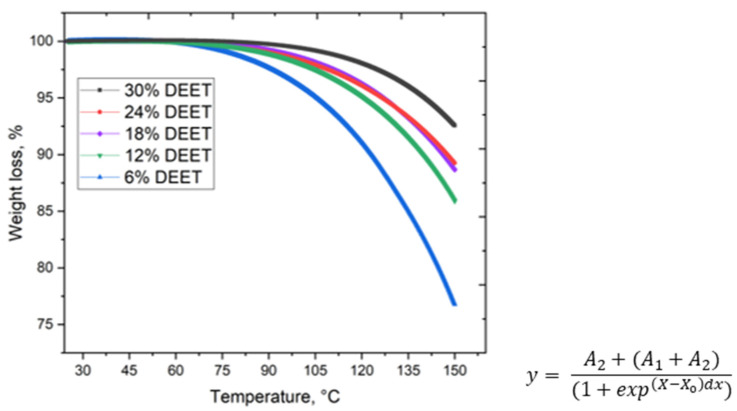
Graph of the weight loss of the precursors of repellent composites with different repellent content as the material is heated.

Thermogravimetric analysis of the repellent composites was carried out. The TGA curves (Figure 4) demonstrated the mass loss of materials at 60 °C, which can be explained by the process of thermal diffusion of the volatile components of the system, since the evaporation of these substances is unlikely due to the high boiling point of each component (189, 197.3 and 288 °C for DMSO, ethylene glycol and DEET, respectively). Moreover, all the TGA curves are described by the Boltzmann equation (R^2^ ≥ 0.999). For each of these equations, the empirical coefficient A_2_ was determined, and the correlation of the obtained data with the values of dynamic viscosity for all compositions was established (Spearman correlation coefficient was +1). Thus, the dynamic viscosity of the samples was shown to be a key factor in the thermal diffusion of the DEET repellent from the developed electroconductive composites.

### 2.5. Determination of N,N-Diethyl-3-methylbenzamide Thermal Diffusion Parameters

**Figure 5:**
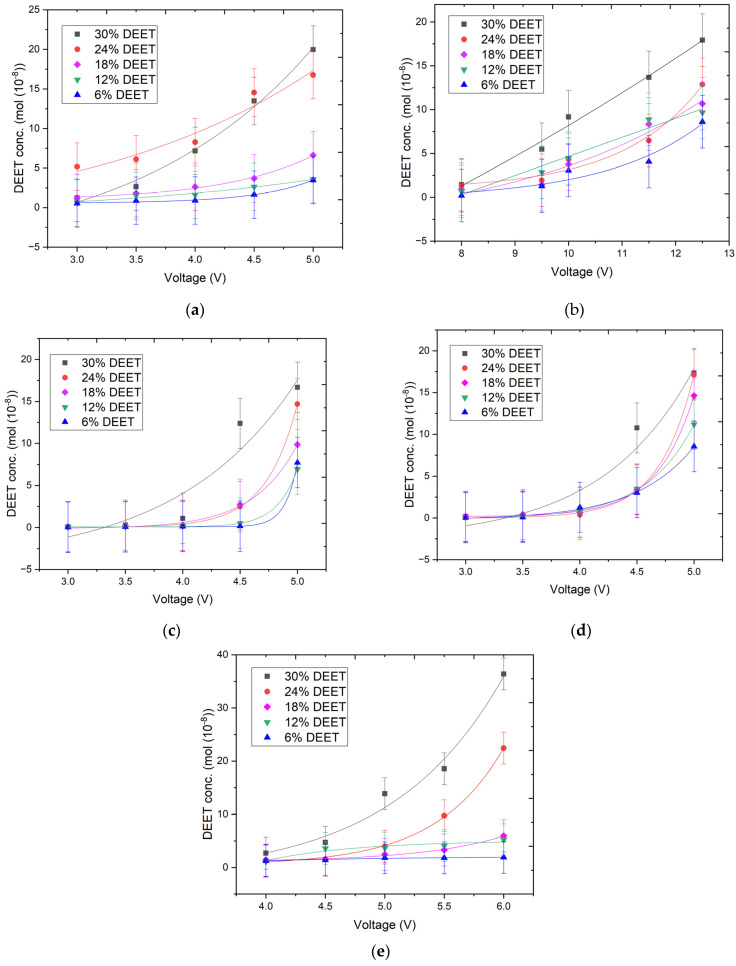
Thermal diffusion plot of DEET with different repellent contents applied in different geometric shapes: straight strip (**a**), meander (**b**), and grids (**c**–**e**).

The thermal diffusion of DEET was studied under the conditions described in the determination of the voltametric properties of repellent composites (Section 3.2.6). The results of the voltametric study of electrically conductive materials were used to determine the intervals of the applied electric voltage. The heating temperatures of each composition, material geometry and voltage were thus determined (Figure 5).

The results of the DEET concentration test by thermal diffusion demonstrated that the repellent composite was heated uniformly in all the cases. This ensured the uniform release of the repellent due to the formation of a temperature gradient. A greater release of repellent (up to 36.4 × 10^−8^ mol) is achieved when the material is applied in a grid **e** shape. In this case, the composite was heated to 116 ± 1 °C. Concurrently, the more intricate application of the material in the meander **b** shape, despite having the longest conductor length (54 mm), also results in the release of DEET from the material at moderate heating, up to 71 ± 1 °C with a concentration of 17.9 × 10^−8^ mol for the composite that contains 30 wt% of repellent. The other application forms of the repellent composite, when subjected to 5 V, exhibited analogous moderate heating, with the DEET concentration reaching 16.7, 17.3 and 20.0 × 10^−8^ mol for grid **c**, grid **b** and straight strip **a** forms, respectively.

**Figure 6:**
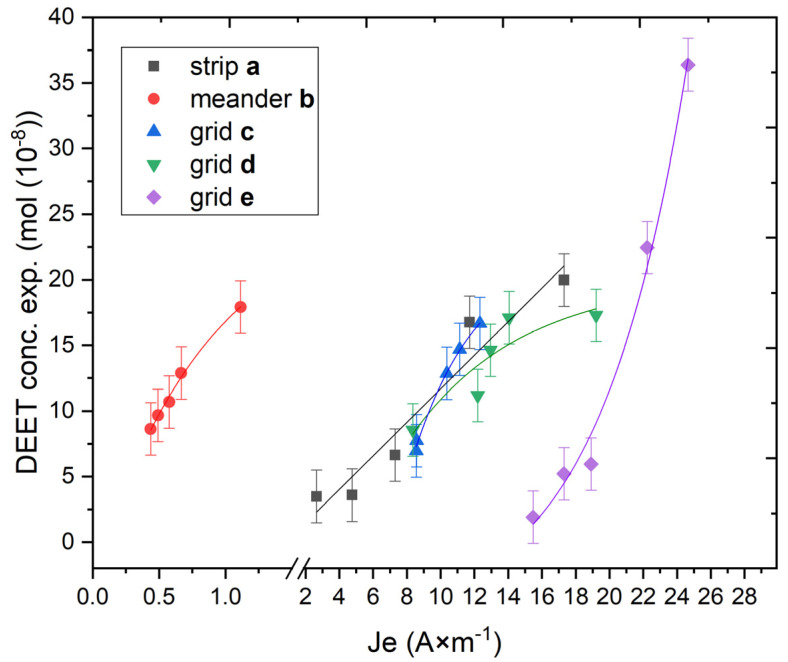
Graph of DEET concentration as a function of electrical flux.

The data obtained as a result of the thermal diffusion of DEET enabled the calculation of the parameters of the electric flux Je (A × m^−1^), which were caused by direct current action on the material and the formation of a temperature gradient in the material. This effect was formulated in the theories of Peltier and Thompson and allows for the calculation of the thermodynamic parameters of a nonequilibrium system [24]. The values of electric flux (Je) for each material in each application form were calculated and plotted as a function of DEET concentration obtained by thermal diffusion (Figure 6) via these laws and the empirical data obtained. Each data point on the graph represents the value of the DEET concentration obtained by thermal diffusion in the materials at the maximum applied voltage (5.6 and 12.5 V) for each application form considered, containing 6–30 wt% of this repellent.

The results demonstrate that, despite the lowest electrical flux value in the meander **b** shape, the concentration of diffusing DEET can be almost the same as that in the straight strip **a**, grid **c** and grid **d** shapes (where the flux value is much higher). This demonstrates that the repellent composite can be used in a wide variety of practical models, achieving the required target concentrations even in complex application geometries. Conversely, the shortest conductor length of the grid **e** shape (14 mm) facilitates the rapid heating of the repellent composite, yielding a substantially higher DEET repellent yield. This configuration is sufficient to operate at an electrical voltage of up to 6 V. In this instance, the value of the electric flux is also the highest. This finding indicates that, according to the provisions of Onsager theory, the general flux of a substance is the highest [25]. Therefore, the most effective way to apply a repellent composite material with a controlled yield of *N*,*N*-diethyl-3-methylbenzamide has been determined.

## 3. Materials and Methods

### 3.1. Materials

*N*,*N*-diethyl-3-methylbenzamide (DEET) was purchased from Merck (Darmstadt, Germany); hydroxyethylcellulose (waterless) was purchased from Alpha Chemika (Mumbai, India); dimethyl sulfoxide (DMSO) was purchased from Aldosa (Moscow, Russia); ethylene glycol was purchased from Acros Organics (Geel, Antwerpen, Belgium); Cu and Ag nanoparticles were purchased from Merck (Darmstadt, Germany); polyaniline (PANI) was purchased from Fisher Scientific (Wien, Austria); activated carbon was purchased from NevaReaktiv (Saint-Petersburg, Russia); multi-walled carbon nanotubes were purchased from Tangfen Tech (Suzhou, China); and acetonitrile was purchased from Honeywell (Charlotte, NC, USA) without further purification. The water was purified via a Direct-Q system Millipore (Darmstadt, Germany).

### 3.2. Methods

#### 3.2.1. Preparation of a Repellent Composite Material

An amount of 0.10 g of hydroxyethyl cellulose (molar weight 736.7 g-mol^−1^) was mixed with 3 g of *N*,*N*-diethyl-3-methylbenzamide and 0.01 g of ethylene glycol. The mixture was subjected to ultrasonication for a period of 30 min in an Elmasonic S 70 H ultrasonic bath (Singen, Germany). Concurrently, 1 g of multi-walled carbon nanotubes was added to 5.89 g of dimethyl sulfoxide, and the mixture is also subjected to ultrasonication for 30 min at a frequency of 37 kHz. Thereafter, both mixtures are mixed with each other.

To obtain formulations with DEET concentrations of 6, 12, 18 and 24 wt% equivalent, the amount of repellent added is reduced while the amount of ethylene glycol is increased (Table 4).

#### 3.2.2. Measurement of the Impedance Spectra of a Repellent Composite Material

The electrochemical impedance spectra (EIS) were measured using an impedance meter Elins Z-2000 (Moscow, Russia). The measurements were conducted within a standard three-electrode cell, with a platinum auxiliary electrode and a silver chloride reference electrode filled with 3.5 mol/L KCl solution. The working electrode was composed of a 3 mm diameter Teflon tube filled with a repellent composite and a current collector that was connected internally. A 2.5 mmol/L solution of K_3_Fe(CN)_6_/K_4_Fe(CN)_6_ in 0.1 mol/L KCl was used as the background solution. A sinusoidal perturbation with an excitation amplitude of 10 mV was applied within the frequency range of 1–1.3∙10^6^ Hz. The impedance hodographs were approximated using Scribner LLC ZView 4 software (Southern Pines, NC, USA).

#### 3.2.3. Measurement of the Voltametric Properties of the Repellent Composite Material

**Figure 7:**
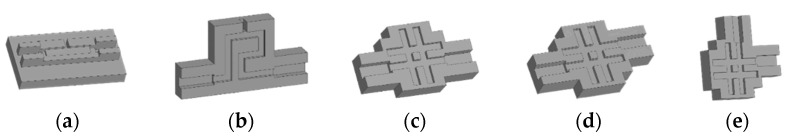
(**a**–**e**). Schematic representation of geometrical models (straight strip (**a**), meander (**b**), and grids (**c**–**e**)) for determination of the voltametric characteristics of composite materials with different content of *N*,*N*-diethyl-3-methylbenzamide.

The voltametric properties of composite materials with different contents of *N*,*N*-diethyl-3-methylbenzamide were determined in models printed on the Creality Ender-5 Pro 3D printer (Shenzhen, China) in the form of a straight strip, meander and grid (layer height of 1 mm) (Figure 7a–e) using the following procedure: the repellent composite was firmly placed into the selected geometric model, and a voltage ranging from 0.5 V to 5 V (or up to 6 V and 12.5 V for the grid **e** and meander **b** models, respectively) was applied to the composite material in 0.5 V steps using a programmable switching laboratory power supply Owon SPE3103 (Zhangzhou, Fujian, China). The output current values (A) were also recorded using a laboratory power supply. The electrical resistivity (R, Ohm) was then determined using an Owon XDM3051 multimeter (Zhangzhou, Fujian, China). In addition, the electrical resistivity values of the composites (ρ, Ohm·m) were calculated.

#### 3.2.4. Measurement of the Rheological Properties of a Repellent Composite Material

The rheological properties of materials with different contents of *N*,*N*-diethyl-3-methylbenzamide were measured using a Anton Paar RheolabQC rheometer (Graz, Austria). During the measurements, the temperature of the samples was maintained at 70 °C ± 1 °C, which is sufficient for the samples to flow within the measuring system without decomposition. The shear rate of the samples was set at 0.5 s^−1^, the number of points was 20, and the measurement interval was 2 s.

#### 3.2.5. Thermogravimetric Analysis (TGA) of a Repellent Composite Material

Thermogravimetric analysis (TGA) (STARe Mettler–Toledo, Columbus, OH, USA) was carried out for five samples with different concentration of DEET. The samples were heated to 150 °C at a heating rate of 10 °C/min under air.

#### 3.2.6. Determination of N,N-Diethyl-3-methylbenzamide Thermal Diffusion Parameters

**Figure 8:**
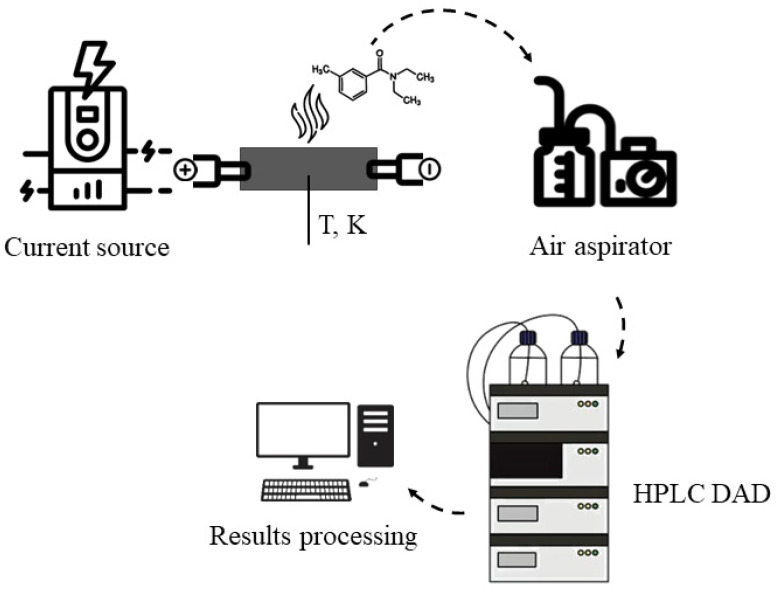
Experiments designed to determine the thermal diffusion parameters of *N*,*N*-diethyl-3-methylbenzamide.

The thermal diffusion parameters of *N*,*N*-diethyl-3-methylbenzamide through the surface of a composite material applied as a thin film were determined according to the following procedure (Figure 8): the repellent composite was placed in a defined geometrical model (straight strip **a**, meander **b** and grids **c**–**e**) and an electric current was applied from different open ends of the model using a programmable switching laboratory power supply Owon SPE3103 (Zhangzhou, Fujian, China) (3 to 5 V for straight strip **a** and grids **c,d**; 4 to 6 V for model grid **e** connections of 8 to 12.5 V; and 5 V for the meander **b** model with a 0.5 V step) (Figure 8). A thermocouple was also connected to the composite material in the middle of each geometric model to record the material heating temperature. After establishing constant values of the output signal (current value (A)) at the power source (for approximately 1 min), an air sample was taken using a Niki-Mlt UOPV 4-40 aspirator (Saint-Petersburg, Russia) with two sequentially connected Petri absorbers, each of which was filled with 10 mL of acetonitrile. Air samples were collected for five minutes at a rate of 2 L per minute. The contents of the absorbents were then evaporated, and the sample was diluted in 5 mL of acetonitrile. The content of *N*,*N*-diethyl-3-methylbenzamide in the samples was determined by high-performance liquid chromatography with diode array detection on an ThermoFisher Ultimate 3000 (Waltham, MA, USA). The concentration of DEET in the samples was calculated by absolute calibration at a wavelength of 210 nm. The DEET solutions used to construct the calibration curves were prepared by diluting the stock solution. The stock solution was prepared by accurately measuring the required amount of compound to the fourth decimal place and diluting it in acetonitrile.

Following the analysis of the obtained data, the DEET concentration values (mol, 10^−8^) were calculated, and their dependence on the flux Je (A∙m^−1^) was plotted using the Peltier and Thompson equation [25,26] (1).

(1) $$J_{e}=L_{e}S_{e}\nabla T,$$

Le—the electrical conductivity of the composite material;
Se—partial entropy of electrons, i.e., heat transferred by electrons;
T—heating temperature of the material, K.

Following the implementation of mathematical transformations on the original Peltier Thompson equation, the subsequent Equation (2) was obtained:

(2) $$J_{e}=\frac{l\cdot I}{V\cdot A}\cdot \alpha \cdot T$$

l— length of the conductor, m;
I—current strength, A;
V—voltage applied to the material, V;
A—cross-sectional area of the geometrical model, m^2^;
α—thermal EMF of multi-walled carbon nanotubes, µV.

Each composite formulation was replicated 5 times in each geometric model.

#### 3.2.7. Data Analysis

Chromatographic data were collected and processed via Chromeleon 7 software (Thermo Fischer Scientific, Waltham, MA, USA). Excel 2019 (Microsoft Corporation, Redmond, WA, USA) and OriginPro 2018 version b9.5.1.195 (Origin Corp., Northampton, MA, USA) were used for detailed calculations and plotting.

## 4. Conclusions

In this study, electrically conductive polymer composites based on *N*,*N*-diethyl-3-methylbenzamide with repellent concentrations of 6–30 wt% were prepared and studied for the first time. The investigation of the voltametric properties of the composite material using impedance analysis demonstrated that the electrical resistivity of the materials is in the range of 150–171 Ohm. This indicates that the developed repellent composite can be utilized when a low voltage (up to 5 and 12.5 V) is applied. In addition, the electrical resistivity of a material depends on both the concentration of repellent and the shape of its application. When more complex geometric shapes of repellent composite materials are used (meander b), it is necessary to apply a higher voltage of up to 12.5 V in order for the material to conduct electric current and heat.

The DEET thermal diffusion study demonstrated that the repellent composite was heated uniformly in all cases, thereby ensuring consistent release of the repellent. The material was applied in the form of grid e, which resulted in greater repellent release (up to 36.4 × 10**^−^**^8^ mol). This can be attributed to the shortest conductor length (14 mm), which facilitated faster heating of the repellent composite. An analysis of the dependence of the DEET concentration on the electrical flux, which was calculated using the Peltier and Thompson equation, revealed that the most efficient form of repellent composite application is grid e. This finding suggests that the general flux of the substance, according to the provisions of the Onsager theory, is the highest. The developed repellent composite represents a promising material for protection against blood-sucking insects and other pests.

## Figures and Tables

**Table 1:** The selection of electroconductive mixture components for the preparation of repellent composite material (n = 10).

Electroconductive Component		Concentration, %		R, Ohm	ρ, Ohm·m
Composite without conductive additives		-		22.5·10^6^	1.8·10^3^
Cu nanoparticles		1.5		12·10^6^	9.4·10^2^
Activated carbon		1		22.5·10^6^	1.8·10^3^
Activated carbon		5		24.5·10^5^	1.9·10^2^
Multi-walled carbon nanotubes	Cu nanoparticles	0.01	1	32.5·10^6^	3.31·10^6^
Multi-walled carbon nanotubes	Cu nanoparticles	0.1	1	22·10^6^	2.24·10^6^
Multi-walled carbon nanotubes	Cu nanoparticles	1	1	52.5·10^5^	0.53·10^6^
Multi-walled carbon nanotubes	PANI/Ag	1.5	3	4.9·10^4^	39.2·10^6^
Multi-walled carbon nanotubes	PANI/Ag	1.5	10	4.95·10^4^	39.6·10^6^
Multi-walled carbon nanotubes		0.01		35·10^6^	3.56·10^6^
Multi-walled carbon nanotubes		0.1		26·10^6^	2.65·10^6^
Multi-walled carbon nanotubes		1		55·10^5^	5.6·10^5^
Multi-walled carbon nanotubes		10		140	0.57

**Table 2:** The electrochemical characteristics of repellent composite materials derived from EIS measurements (n = 10).

DEET Content in the Sample, wt%.	Rs, (Ohm)	Rp, (Ohm)	CPE1 (μ S)	χ^2^
6	71.27	150.2	0.42638	0.0005
12	56.47	159.0	0.34294	0.0003
18	153.0	152.4	0.57981	0.0003
24	186.0	154.6	0.68429	0.0004
30	53.18	171.0	0.52097	0.0008

Rs—solution resistivity; Rp—charge transfer resistivity; CPE1—constant phase element; and χ^2^—convergence criterion.

**Table 3:** Electrical resistivity of the repellent composites in different applications as a function of the DEET content of the sample (n = 10).

DEET Concentration, wt%.	Output Current Value (A) at Maximum Voltage Current				
	Form of Composite Application (Conductor Length)				
	Straight Strip a (16 mm)	Grid d (20 mm)	Grid e (14 mm)	Grid c (16 mm)	Meander b (54 mm)
30	0.121	0.071	0.060	0.052	0.020
24	0.091	0.055	0.055	0.048	0.013
18	0.056	0.053	0.050	0.047	0.011
12	0.038	0.050	0.045	0.045	0.009
6	0.022	0.037	0.041	0.043	0.008

**Table 4:** Preparation of repellent composite material with different DEET content.

Substance	Composite with 30 wt% DEET	Composite with 24 wt% DEET	Composite with 18 wt% DEET	Composite with 12 wt% DEET	Composite with 6 wt% DEET
Hydroxyethylcellulose	0.10 g				
Dimethyl sulfoxide	5.89 g				
Multi-walled carbon nanotubes	1 g				
*N*,*N*-diethyl-3-methylbenzamide (DEET)	3 g	2.4 g	1.8 g	1.2 g	0.6 g
Ethylene glycol	0.01 g	0.61 g	1.21 g	1.81 g	2.41 g

## Data Availability

The manuscript reports the complete dataset. If needed, the corresponding author can be contacted via email for further calculations.

## Abbreviations

DEET	*N*,*N*-diethyl-3-methylbenzamide
TGA	Thermogravimetric analysis
PANI	Polyaniline
DMSO	Dimethyl sulfoxide
EIS	Electrochemical impedance
EEC	Equivalent electrical circuit
CPE1	Constant phase element
HPLC DAD	High-performance liquid chromatography with diode array detection
